# Crystal Structure of the Open State of the *Neisseria gonorrhoeae* MtrE Outer Membrane Channel

**DOI:** 10.1371/journal.pone.0097475

**Published:** 2014-06-05

**Authors:** Hsiang-Ting Lei, Tsung-Han Chou, Chih-Chia Su, Jani Reddy Bolla, Nitin Kumar, Abhijith Radhakrishnan, Feng Long, Jared A. Delmar, Sylvia V. Do, Kanagalaghatta R. Rajashankar, William M. Shafer, Edward W. Yu

**Affiliations:** 1 Department of Chemistry, Iowa State University, Ames, Iowa, United States of America; 2 Department of Physics and Astronomy, Iowa State University, Ames, Iowa, United States of America; 3 Bioinformatics and Computational Biology Interdepartmental Graduate Program, Iowa State University, Ames, Iowa, United States of America; 4 NE-CAT and Department of Chemistry and Chemical Biology, Cornell University, Argonne National Laboratory, Argonne, Illinois, United States of America; 5 Department of Microbiology and Immunology, Emory University School of Medicine, Atlanta, Georgia, United States of America; 6 Laboratories of Microbial Pathogenesis, VA Medical Center, Decatur, Georgia, United States of America; Arizona State University, United States of America

## Abstract

Active efflux of antimicrobial agents is one of the most important strategies used by bacteria to defend against antimicrobial factors present in their environment. Mediating many cases of antibiotic resistance are transmembrane efflux pumps, composed of one or more proteins. The *Neisseria gonorrhoeae* MtrCDE tripartite multidrug efflux pump, belonging to the hydrophobic and amphiphilic efflux resistance-nodulation-cell division (HAE-RND) family, spans both the inner and outer membranes of *N. gonorrhoeae* and confers resistance to a variety of antibiotics and toxic compounds. We here describe the crystal structure of *N. gonorrhoeae* MtrE, the outer membrane component of the MtrCDE tripartite multidrug efflux system. This trimeric MtrE channel forms a vertical tunnel extending down contiguously from the outer membrane surface to the periplasmic end, indicating that our structure of MtrE depicts an open conformational state of this channel.

## Introduction

*Neisseria gonorrhoeae* is a Gram-negative diplococcus, which is found only in humans and causes the sexually transmitted disease gonorrhea. Gonorrhea is one of the oldest described diseases; however, it remains a significant global problem with more than 100 million cases reported annually worldwide and antibiotic resistance is a major concern [1]. Since *N. gonorrhoeae* is a strictly human pathogen and can colonize both male and female genital mucosal surfaces and other sites, it has developed mechanisms to overcome antimicrobial systems of the host's innate defense. One major mechanism that this bacterium uses to repel antimicrobial agents is the expression of multidrug efflux pumps that recognize and actively export a variety of structurally unrelated toxic compounds from the bacterial cell, including antibacterial peptides, long-chain fatty acids, and several clinically important antibiotics [2]–[5].

The best characterized and most clinically important efflux system in *N. gonorrhoeae* is the MtrCDE tripartite multidrug efflux system [6]–[8], which belongs to the hydrophobic and amphiphilic efflux resistance-nodulation-cell division (HAE-RND) family. In Gram-negative bacteria, efflux systems of the HAE-RND family play major roles in the intrinsic and acquired tolerance of antibiotics and toxic compounds [9]. They represent key components for Gram-negative pathogens to use in overcoming toxic environments unfavorable for their survival. Typically, an RND efflux pump [10]–[16] works in conjunction with a periplasmic membrane fusion protein [17]–[20], and an outer membrane channel to form a functional protein complex [21], [22]. The resulting tripartite efflux system spans the inner and outer membranes of Gram-negative bacteria to export substrates directly out of the cell [9].

For the MtrCDE tripartite efflux system, MtrD [7], [23] is a large proton-motive-force (PMF)-dependent inner membrane HAE-RND efflux pump composed of 1,067 amino acids. MtrE [24], [25] is a 447 amino acid protein that forms an outer membrane channel. The membrane fusion protein MtrC [25], [26], containing 412 amino acids, bridges MtrD and MtrE to form the tripartite efflux complex MtrCDE. This powerful efflux complex spans the entire cell envelope of *N. gonorrhoeae* and mediates the export of hydrophobic antimicrobial agents, such as antibiotics, nonionic detergents, antibacterial peptides, bile salts and gonadal steroidal hormones [2], [7], [27], [28].

Currently, there are only two crystal structures of HAE-RND efflux pumps resolved by crystallography. These efflux pumps are the *Escherichia coli* AcrB [10]–[15] and *Pseudomonas aeruginosa* MexB [16] multidrug transporters. The crystal structures of the other components of these tripartite complex systems have also been determined. These include the outer membrane channels *E. coli* TolC [21] and *P. aeruginosa* OprM [22] as well as the periplasmic membrane fusion proteins *E. coli* AcrA [19] and *P. aeruginosa* MexA [20]–[22]. In Gram-negative bacteria, several other crystal structures of outer membrane channels, such as VceC of *Vibro choleria* [29] and CusC of *E. coli* [30], [31], have also been reported.

Thus far, there is no structural information available for any protein component of the MtrCDE tripartite complex system. However, it has been reported that individual protein components of this tripartite system are able to interact with each other, suggesting that the tripartite MtrCDE pump is assembled in the form of MtrD_3_-MtrC_6_-MtrE_3_ [25]. It is important to note that this result is indeed in good agreement with the CusBA adaptor-transporter co-crystal complex [32], [33] of the CusCBA efflux system [30], [31], [34]–[37] where the stoichiometry is 6:3 adaptor-to-transporter molar ratio [32], [33].

Here we present the crystal structure of the outer membrane MtrE channel, which represents an open conformational state of this multidrug efflux protein. The structure suggests that the interior surface of the channel protein forms a continuous, elongated tunnel, which extends from the outer membrane surface and leads down to the tip of the α-helical periplasmic domain. In addition, an aspartate ring created by six aspartates is found at the tip of the periplasmic tunnel, presumably acting as a selectivity gate of the channel.

## Results and Discussion

### Overall structure of the *N. gonorrhoeae* MtrE outer membrane channel

**Figure 1:**
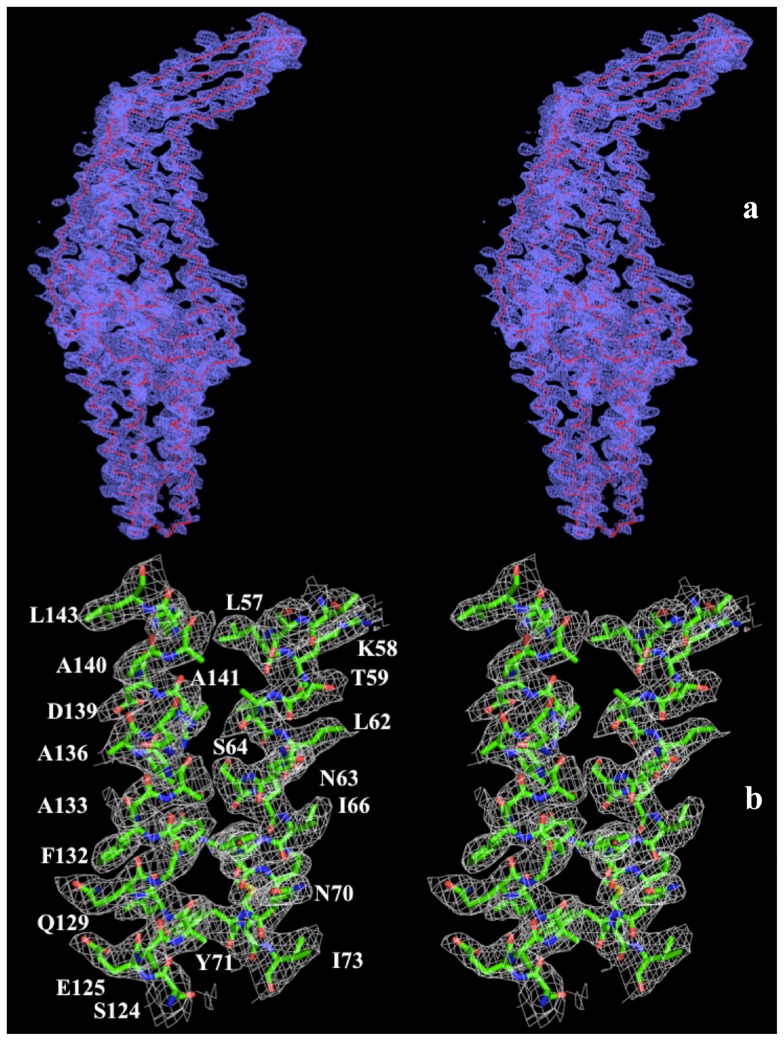
Stereo view of the composite omit electron density map of the MtrE channel protein at a resolution of 3.29 Å. (a) The composite omit map contoured at 1.2 σ is in blue. The Cα traces of MtrE are in red. (b) Representative section of the electron density at the interface between H2 and H3 of the periplasmic domain of MtrE. The electron density (colored white) is contoured at the 1.2 σ level and superimposed with the final refined model (green, carbon; red, oxygen; blue, nitrogen).

**Figure 2:**
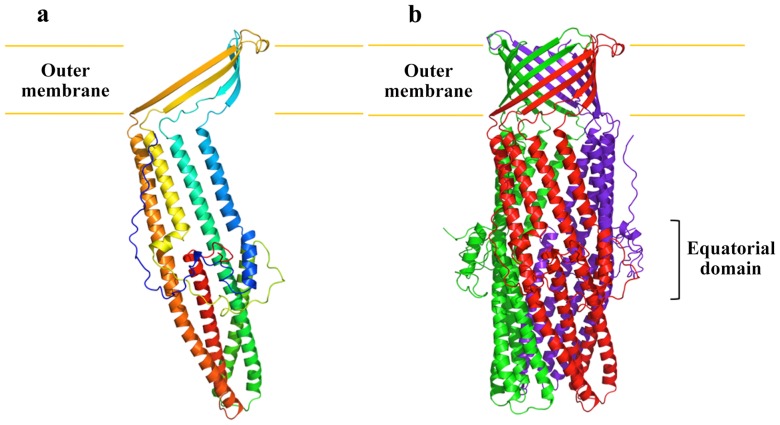
Structure of the *N. gonorrhoeae* MtrE channel protein. (a) Ribbon diagram of a protomer of MtrE viewed in the membrane plane. The molecule is colored using a rainbow gradient from the N-terminus (blue) to the C-terminus (red). (b) Ribbon diagram of the MtrE trimer viewed in the membrane plane. Each subunit of MtrE is labeled with a different color.

We cloned, expressed, and purified the full-length MtrE outer membrane channel containing a 6×His tag at the C-terminus. We obtained crystals of this membrane protein using vapor diffusion. We then used molecular replacement, utilizing the structure of *P. aeruginsa* OprM (pdb code: 1WP1) [22] to determine the three-dimensional structure of MtrE. The diffraction data were indexed to the space group *P*6_3_22. Data collection and refinement statistics are summarized in Table 1. The resulting electron density maps (Fig. 1) reveal that the asymmetric unit consists of one protomer. The crystal structure of the full-length MtrE outer membrane channel protein was then determined to a resolution of 3.29 Å (Table 1). The final model comprises 99% of the total amino acids (residues 1–445) (Fig. 2a). The final structure is refined to R_work_ and R_free_ of 24.1% and 29.4%, respectively. Superimposition of the final structure of MtrE with that of OprM (pdb code: 1WP1) [22] results in a RMSD of 18.2 Å over 445 C^α^ atoms, suggesting highly significant difference in the overall tertiary structures between these two channel proteins (Fig. S1).

**Table 1:** Data collection and refinement statistics.

Data set	MtrE
**Data Collection**	
Wavelength (Å)	0.98
Space group	*P*6_3_22
Resolution (Å)	50–3.29
	(3.41–3.29)
Cell constants (Å)	
a	93.89
b	93.89
c	391.54
α, β, γ (°)	90, 90, 120
Molecules in ASU	1
Redundancy	3.4 (3.3)
Total reflections	287,882
Unique reflections	16,706
Completeness (%)	98.7 (95.6)
R_sym_ (%)	11.8 (43.5)
R_pim_ (%)	7.6 (30.2)
Average I/σ(I)	9.7 (2.4)
**Refinement**	
Resolution (Å)	50–3.29
R_work_	24.1
R_free_	29.4
rms deviation from ideal	
bond lengths (Å)	0.009
bond angles (°)	1.249
**Ramachandran**	
most favoured (%)	96.8
additional allowed (%)	3.2
generously allowed (%)	0.0
disallowed (%)	0.0

**Figure 3:**
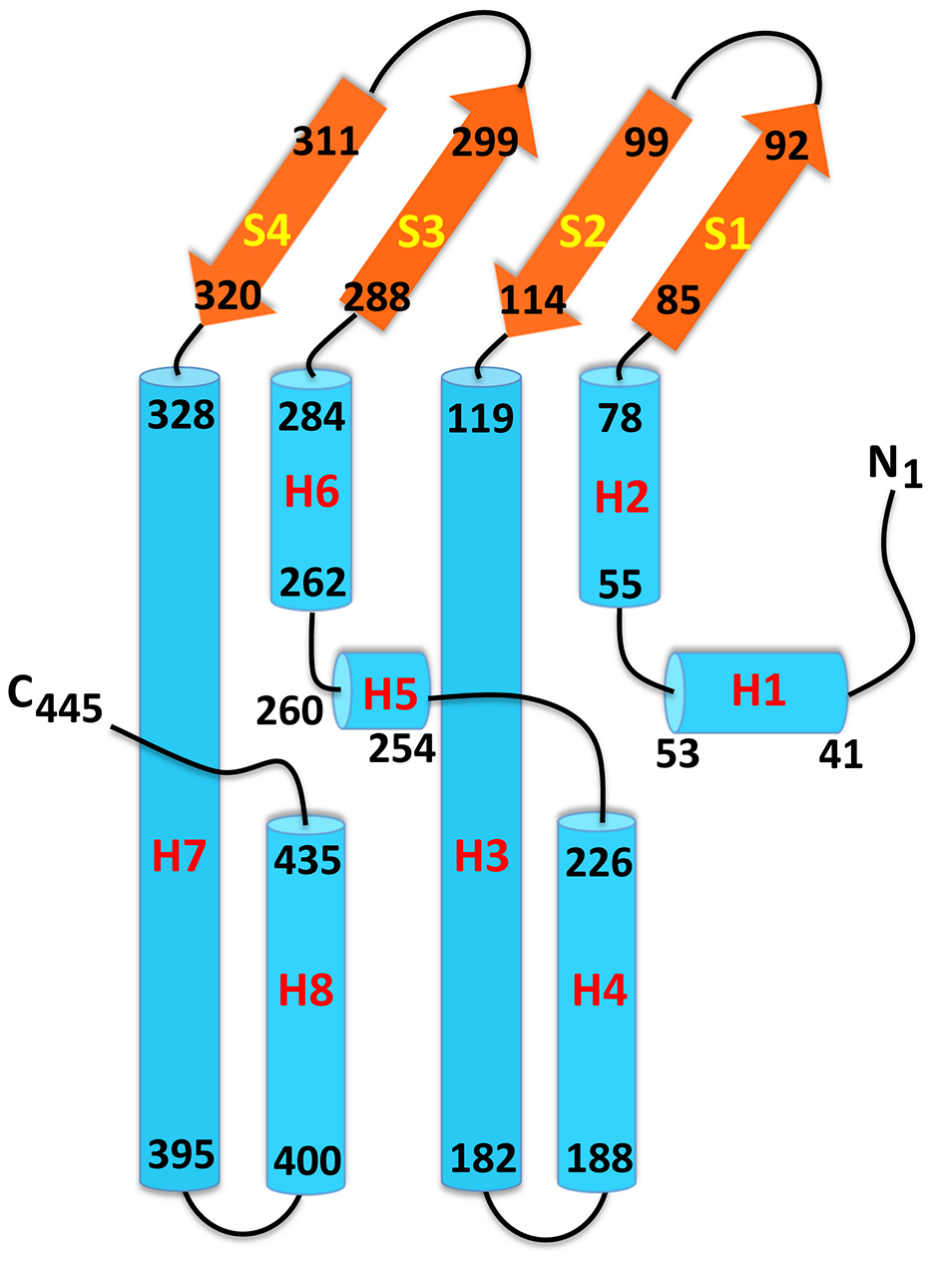
Secondary structural topology of the MtrE monomer. The topology was constructed based on the crystal structure of MtrE. The α-helices and β-strands are colored cyan and orange, respectively.

Like TolC [21] and OprM [22], MtrE exists as a homotrimer that forms a ∼130 Å long α/β barrel (Fig. 2b). Each subunit of MtrE contains four β-strands (contributing to the 12-stranded outer membrane β-barrel) and eight α-helices (forming the elongated periplasmic α-barrel) (Fig. 3). These four β-strands (S1, S2, S3 and S4) constitute the β-barrel domain and are organized in an antiparallel fashion, spanning the outer membrane. In contrast, the elongated periplasmic tunnel of MtrE contains six α-helices. Similar to the structure of TolC, two long helices (H3 and H7) are found to extend across the entire length of the periplasmic α-helical tunnel. The α-helical tunnel of MtrE also includes two pairs of shorter α-helices, (H2 and H4) and (H6 and H8). These two pairs of shorter helices stack end-to-end to form pseudocontinuous helices, which contribute coiled-coil interactions with the two long helices. The equatorial domain of MtrE is composed of two helices (H1, H5) and the remaining elements at this domain are mostly unstructured. The periplasmic tunnel of MtrE is ∼100 Å long with an outermost diameter of ∼35 Å at the tip of the tunnel.

### The crystal structure of MtrE shows that the internal surface of the protein forms a continuous channel

**Figure 4:**
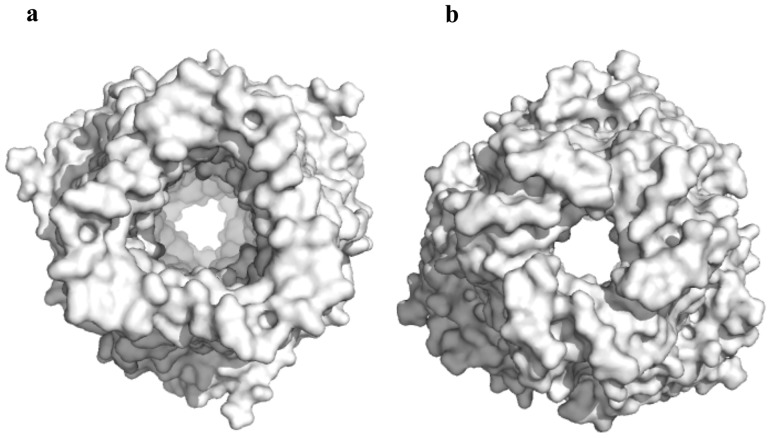
Surface representations of the trimeric MtrE channel. The views from both the (a) extracellular and (b) periplasmic sides suggest that the MtrE channel is in its open form.

In view of the crystal structure of MtrE, it is found that the internal surface of the protein forms a continuous channel. This channel is completely open and fully accessible through both the periplasmic end and outer membrane surface, suggesting that the MtrE channel is at its open conformational state. To date, most of the available structures of outer membrane channels, including TolC [21], OprM [22] and CusC [30], [31] are closed at one or both sides. However, several structures of the TolC mutants, which led to the opening of the TolC exit duct, have been reported [38], [39]. These structures also indicate how important residues interact with one another to control the opening and closing of the periplasmic end of this channel. Nonetheless, our crystal structure of the wild-type MtrE channel indicates that this channel is in its open conformational state (Fig. 4). The widest section of the channel is located at the surface of the outer membrane, with the internal diameter of ∼22 Å. The volume of the continuous channel formed by the internal surface of the MtrE trimer is ∼45,000 Å^3^.

**Figure 5:**
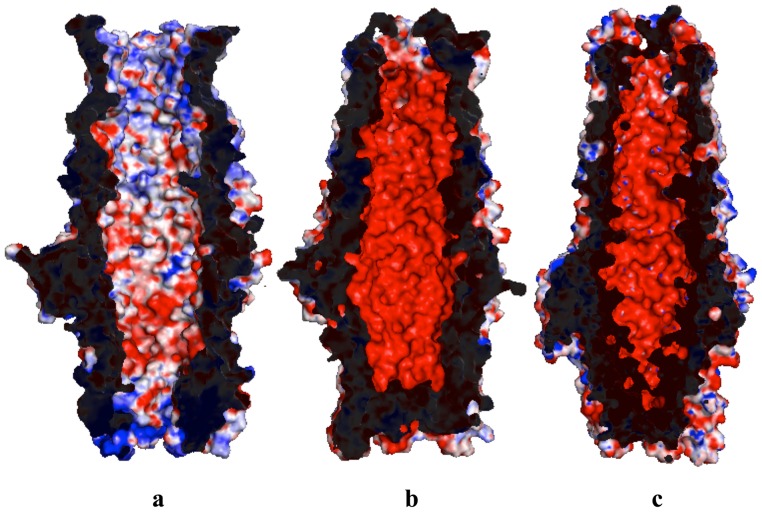
Electrostatic surface potentials of MtrE, TolC and OprM. Surface representations of the inside of the (a) MtrE, (b) TolC (pdb id: 1EK9) [21] and (c) OprM (pdb id: 1WP1) [22] channels colored by charge (red, negative −15 kT/e; blue, positive +15 kT/e).

In addition, all available structures of outer membrane channel proteins, such as TolC [21], OprM [22] and CusC [30], [31] indicate that the interior surfaces of these channels are highly electronegative. However, MtrE is distinct in that its internal surface does not have extensive positively or negatively charged patches (Fig. 5). On the contrary, the charge distribution of the outside surface of MtrE is very similar to other outer membrane channels, in which the outside surfaces of all these channels have no extensive charged patches.

### The interior aspartate ring

**Figure 6:**
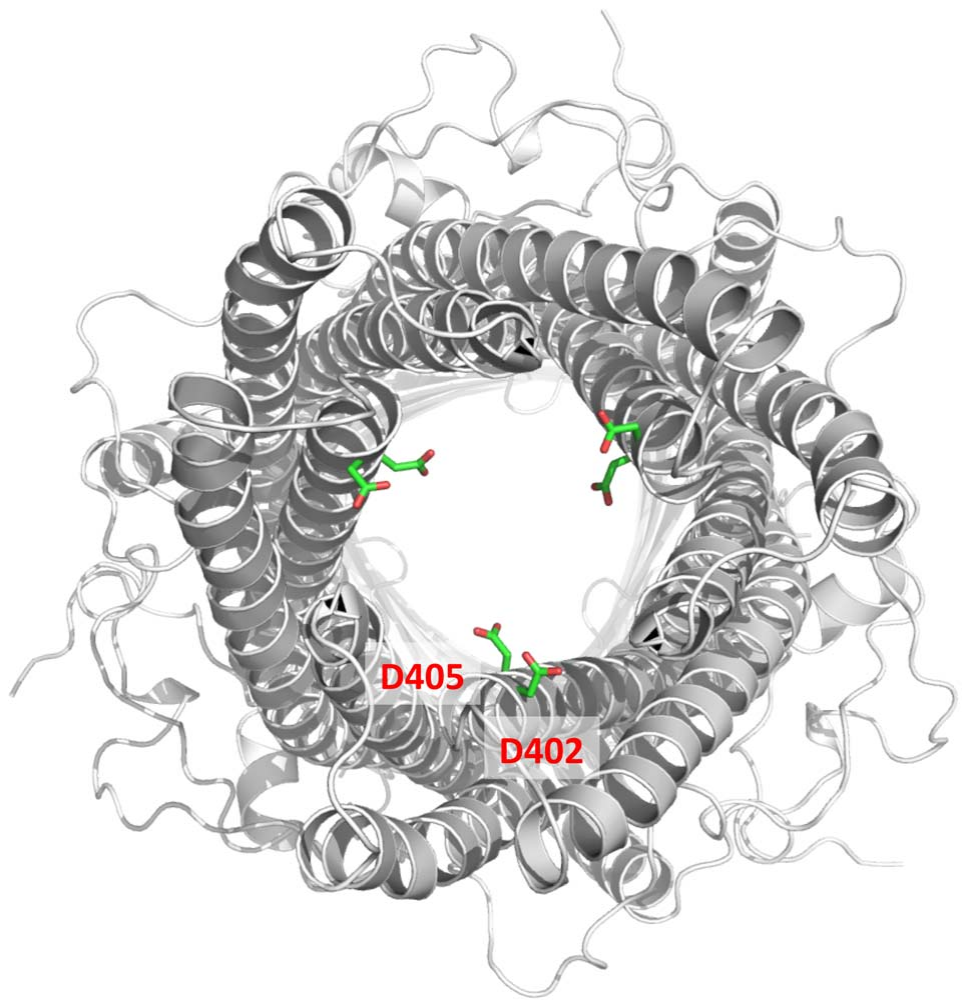
The periplasmic aspartate ring. Viewed from the periplasmic side, this aspartate ring (formed by D402 and D405 of each protomer) is found at the periplasmic entrance of the interior of the MtrE channel. It is likely that this ring is responsible for the selectivity of the channel.

Like the TolC channel, an aspartate ring is found at the periplasmic entrance of the interior of the MtrE channel. Each protomer of MtrE contributes D402 and D405 to form two concentric circles of negative charges in the inner cavity of the trimeric MtrE channel (Fig. 6). Thus, this interior aspartate ring is composed of six aspartate residues. In TolC, the corresponding aspartate ring creates a selectivity gate for this channel and this ring can be blocked by large cations. Fig. S2 illustrates the alignment of protein sequences of the MtrE and TolC channels. The internal diameter of the MtrE aspartate ring is ∼12 Å, which creates the narrowest region of the tunnel. It is likely that this aspartate ring is responsible for the selectivity of the channel, similar to the case of TolC [40]. Indeed, it has been demonstrated that the aspartate ring of MtrE can be blocked by the large positively charged hexamminecobalt (III) complex [25].

In comparison with the TolC structures, the apparent dilation of our MtrE structure is higher than any reported structure of the open state of TolC. For example, the longest distances between D374 residues of the three TolC protomers in the TolC^WT^, TolC^RS^ and TolC^YFRS^ structures, measured between the side chain O(δ2) atoms, are 6.2, 6.2 and 9.9 Å [39]. The longest distances between the Cα atoms of D374 residues within these TolC trimers are 11.7, 12.3 and 15.4 Å [39]. In the MtrE trimer, the corresponding distance between the side chain O(δ2) atoms of D405 residues becomes 11.8 Å. Additionally, the distance between the Cα atoms of these aspartates is 16.0 Å. It is suspected that the open conformational state of MtrE reflects the low-pH form of this channel, as we crystallized this channel at low pH which would neutralize the aspartate ring.

During the course of substrate import or export, the aspartate ring may need to dilate and increase its internal diameter to allow substrates to pass through the channel. Although our structure indicates that MtrE is capable of opening this channel by itself, it has been suggested that the dilation and constriction of the aspartate ring may be controlled by the MtrC periplasmic membrane fusion protein [24]. In addition, it has been observed that the MtrE channel is able to allow the large vancomycin molecule to enter the cell [24]. However, it only does so in response to the binding of MtrC [24], presumably enhancing the degree of dilation of the MtrE channel. It appears that the opening and closing of the MtrE channel may be induced by the change in conformation of the MtrC membrane fusion protein, which propagates the progressive motion of the MtrD multidrug efflux pump within the transport cycle to the MtrE channel. As MtrD is a PMF-dependent pump, this may imply that active proton translocation within the MtrD inner membrane efflux pump provides the energy to open and close the MtrE outer membrane channel.

### The exterior intra- and inter-protomer grooves

The outermost surface of the periplasmic domain of the MtrE trimer forms three intra-protomer and three inter-protomer grooves. These grooves are likely to provide interaction sites for the MtrC membrane fusion protein. There is a chance that the α-helical coiled-coil domain of MtrC could fit into these grooves and contact MtrE to function. Based on the co-crystal structure of the CusBA adaptor-transporter complex [32], [33], the β-barrel domains of the elongated MtrC membrane fusion protein should interact with the periplasmic domain of the MtrD pump, bridging the gap between the MtrD and MtrE membrane proteins. This suggests that MtrC could relay conformational changes from the MtrD pump to MtrE channel, allowing these two efflux proteins to communicate with each other. In turn, this relay network may control the opening and closing of MtrE.

Several surface-exposed residues, including E161, R168, E407, E414 and Q421, are found at the intra-protomer groove of MtrE. Interestingly, many of these residues are charged amino acids. Likewise, a number of charged and polar residues, such as Q167, N178, E198, E202, R215 and R219, also line the surface of the inter-protomer groove of MtrE. These charged and polar residues may be critical for MtrE-MtrC interaction. Indeed, it has been identified that residues E414 and Q421 (found in the intra-protomer groove) as well as N178 (located at the inter-protomer groove) are important for the function of the MtrCDE tripartite efflux pump [24].

The α-helical hairpin of the MtrC membrane fusion protein may directly engage the inter- and intra-protomer grooves of the MtrE channel to form a complex. Thus, these surface-exposed charged and polar residues, found within the inter- and intra-protomer grooves of MtrE, may be crucial for the binding of MtrC to the MtrE channel. Exactly how MtrC and MtrE interact must await confirmation by elucidation of the crystal structure of the MtrC membrane fusion protein.

It is well established that overexpression of RND multidrug efflux pumps leads to a resistant phenotype in pathogenic organisms. Because of the fact that these multidrug efflux pumps are able to respond to a wide spectrum of substrates, pathogenic bacteria that overexpress them can be selected for by many different agents. Thus, it is very important to understand the molecular mechanism as well as detailed structural information of these efflux pumps in order to combat infectious diseases. The control of gonorrhea has been compromised by the increasing proportion of infections due to antibiotic-resistant strains, which are growing at an alarming rate. The availability of the crystal structure of the MtrE efflux channel may allow us to rationally design agents that block its function and eventually heighten the sensitivity of *N. gonorrhoeae* to antimicrobials.

## Methods

### Cloning, expression and purification of the outer membrane MtrE channel

Briefly, the full-length MtrE membrane protein containing a 6×His tag at the C-terminus was overproduced in *E. coli* C43(DE3) cells possessing the expression vector pBAD22bΩ*mtrE*. Cells were grown in 12 L of LB medium with 100 μg/ml ampicillin at 37°C. When the OD_600_ reached 0.5, the culture was cooled down to 25°C and then treated with 0.2% (w/v) arabinose to induce *mtrE* expression. Cells were harvested after shaking for 16 h at 25°C. The collected bacteria were resuspended in buffer containing 20 mM Na-HEPES (pH 7.5), 300 mM NaCl and 1 mM PMSF, and then disrupted with a French pressure cell. The membrane fraction was collected by ultracentrifugation, followed by a pre-extraction procedure by incubating in buffer containing 0.5% (w/v) sodium lauroyl sarcosinate, 20 mM Na-HEPES (pH 7.5) and 50 mM NaCl for 0.5 h at room temperature. The outer membrane was collected and washed twice with buffer containing 20 mM Na-HEPES (pH 7.5) and 50 mM NaCl. The MtrE membrane protein was then solubilized in 2% (w/v) n-dodecyl β-D-maltoside (DDM). Insoluble material was removed by ultracentrifugation at 100,000×g. The extracted protein was purified with a Ni^2+^-affinity column. The purity of the MtrE protein (>95%) was judged using 12% SDS-PAGE stained with Coomassie Brilliant Blue. The purified protein was then dialyzed and concentrated to 15 mg/ml in buffer containing 20 mM Na-HEPES (pH 7.5), 200 mM NaCl and 0.05% (w/v) DDM.

### Crystallization of MtrE

Crystals of the 6×His MtrE were obtained using sitting-drop vapor diffusion. A 2 μl protein solution containing 15 mg/ml MtrE protein in 20 mM Na-HEPES (pH 7.5), 200 mM NaCl and 0.05% (w/v) DDM was mixed with a 2 μl of reservoir solution containing 20% PEG 400, 0.2 M sodium acetate (pH 4.6), 0.25 M MgSO_4_ and 2% (w/v) n-octyl-β-D-glucoside (OG). The resultant mixture was equilibrated against 500 μl of the reservoir solution at room temperature. Crystals of MtrE grew to a full size in the drops within two weeks. Typically, the dimensions of the crystals were 0.2 mm ×0.2 mm ×0.2 mm. Crystals were flash-cooled, using solution containing 30% PEG 400, 0.2 M sodium acetate (pH 4.6), 0.25 M MgSO_4_, 0.05% DDM and 2% OG, as a cryoprotectant before data collection.

### Data collection, structural determination and refinement

All diffraction data were collected at 100K at beamline 24ID-C located at the Advanced Photon Source, using an ADSC Quantum 315 CCD-based detector. Diffraction data were processed using DENZO and scaled using SCALEPACK [41].

Crystals of the MtrE channel protein belong to the space group *P*6_3_22 (Table 1) and the best crystal diffracted x-ray to a resolution of 3.29 Å. Analysis of Matthew's coefficient indicated the presence of one MtrE protomer (49.29 kDa) per asymmetric unit, with a solvent content of 75.8%.

The structure of MtrE was phased using molecular replacement, utilizing the structure of OprM (pdb id: 1WP1) [22] as a search model. After tracing the initial model manually using the program Coot [42] the model was refined against the data at 3.29 Å-resolution using TLS refinement techniques adopting a single TLS body as implemented in PHENIX [43] leaving 5% of reflections in Free-R set. Iterations of refinement using PHENIX [43] and CNS [44] and model building in Coot [42] lead to the current model, which contains 455 amino acids with excellent geometrical characteristics (Table 1).

### Accession code

Atomic coordinates and structure factors have been deposited with the Protein Data Bank under the accession code 4MT0.

## Supporting Information

Figure S1 **Comparison of the structures of the MtrE and OprM channels.** This is a superimposition of a subunit of MtrE (red) onto that of OprM (blue), indicating that the structures of these two efflux pumps are quite distinct.
(TIFF)

Figure S2 **Alignment of the amino acid sequences of the MtrE and TolC channels.** The alignment was done using CLUSTAL W (*, identical residues; :, >60% homologous residues). Sequence alignment indicates that these two outer membrane channels share 16.7% identity.
(TIFF)
