# Supplementary figures and images for: Crystal Structure of the Open State of the Neisseria gonorrhoeae MtrE Outer Membrane Channel
Source: PLoS One. 2014 Jun 5;9(6):e97475. doi: 10.1371/journal.pone.0097475 (PMC4046963; doi:10.1371/journal.pone.0097475)

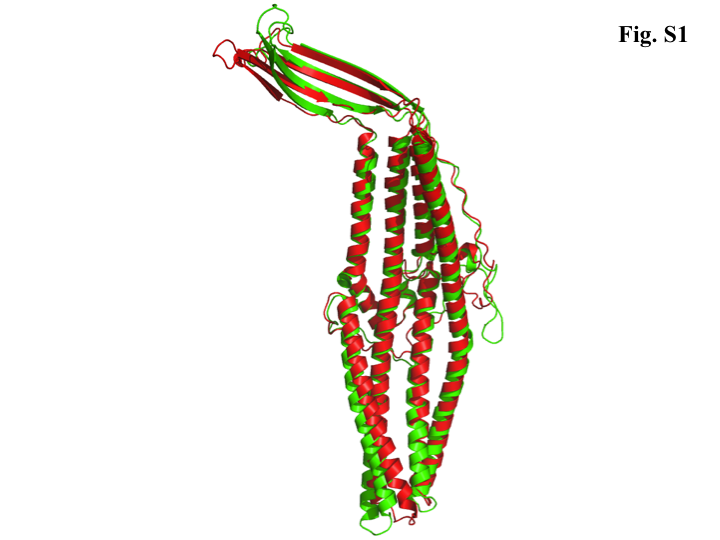

Supplement: Figure S1 — Comparison of the structures of the MtrE and OprM channels. This is a superimposition of a subunit of MtrE (red) onto that of OprM (blue), indicating that the structures of these two efflux pumps are quite distinct. (TIFF) [file pone.0097475.s001.tiff]

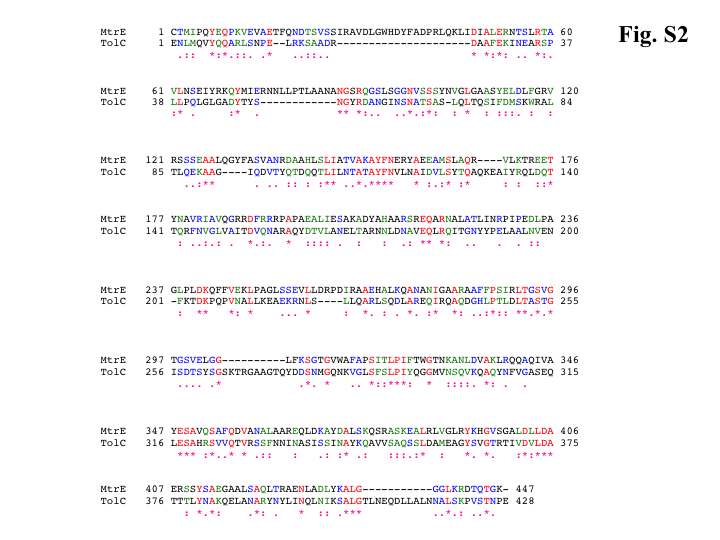

Supplement: Figure S2 — Alignment of the amino acid sequences of the MtrE and TolC channels. The alignment was done using CLUSTAL W (*, identical residues; :, >60% homologous residues). Sequence alignment indicates that these two outer membrane channels share 16.7% identity. (TIFF) [file pone.0097475.s002.tiff]
